# *De Qi*, a Threshold of the Stimulus Intensity, Elicits the Specific Response of Acupoints and Intrinsic Change of Human Brain to Acupuncture

**DOI:** 10.1155/2014/914878

**Published:** 2014-07-06

**Authors:** Dai-Shi Tian, Jin Xiong, Qing Pan, Fang Liu, Lu Wang, Sha-Bei Xu, Guang-Ying Huang, Wei Wang

**Affiliations:** ^1^Department of Neurology, Tongji Hospital, Tongji Medical College, Huazhong University of Science and Technology, Wuhan 430030, China; ^2^Institute of Integrated Traditional and Western Medicine, Tongji Hospital, Tongji Medical College, Huazhong University of Science and Technology, Wuhan 430030, China

## Abstract

*Objectives.* *De qi* is the subjective constellation of sensations perceived by the acupuncturists and patients as described in several literatures, but the absence of quantitative evaluation methods in *de qi* restricts the use of acupuncture treatment widely in the world. In the present study, we tried to investigate the intrinsic property of *de qi* is and how evaluate it quantitatively. *Methods.* 30 healthy adult volunteers were determined to investigate intrinsic changes in the human body after acupuncture with *de qi*. *Results.* Acupuncture treatment with *de qi* apparently increased acupoint blood flow, tissue displacement, and the amplitude of myoelectricity after *de qi* on acupoints. Furthermore, acupuncture treatment induced fMRI signal increase/decrease in different brain regions although no significant change in electroencephalography. *Interpretation.* The intrinsic change of the subjects representing the specific response of acupoints and human brain to acupuncture indicated that *de qi* might be evaluated quantitatively by those above aspects, which facilitated the confirmation in validity and propagation of this treatment modality widely in the world.

## 1. Introduction

Now, acupuncture is becoming increasingly popular in the world and is routinely recommended for the treatment of pain and for relief of many other symptoms such as nausea and vomiting associated with chemotherapy, substance dependency, and chronic disorders difficult to manage with conventional treatment [1]. In women's health, acupuncture has been found to be beneficial for patients with premenstrual syndrome, dysmenorrhea, and several pregnancy-related conditions [2]. Whatever the exact pathway may have been, by the time traditional Chinese medicine was codified at some time in the first century BC (in a canonical text known as the* Inner Classic of the Yellow Emperor*), acupuncture was already a signature therapy of Chinese medicine.* De qi*, achieving* qi*, which is interpreted as the flow of “vital energy,” is the resultant effect of characteristic needle manipulation, sensations perceived by the patients, which manifests as numbness, heaviness, distention, and soreness, with spreading sensation; and it is also perceived by the acupuncturists, which manifests as heavy and tight sensation coming from beneath the needle [3]. Although the underlying therapeutic mechanism remains unclear, it is generally accepted that “*de qi*” is the sign of optimal effect of needle manipulation, and more importantly,* de qi* is considered as the sine qua non of acupuncture for the achievement of a clinical therapeutic effect according to traditional Chinese medicine (TCM) [4–8]. There is a long-held belief in the traditional theory and clinical practice of acupuncture that the intensity of the stimulus must reach a threshold to elicit* de qi*, which plays a pivotal role in achieving the best therapeutic effects. Recently, our study was published in CMAJ and found evidence that acupuncture with* de qi* improved facial muscle recovery, disability, and quality of life among patients with Bell palsy. Stronger intensity of* de qi* was associated with better therapeutic effects [9]. However, the absence of quantitative evaluation methods in* de qi* restricts the use of acupuncture treatment widely in the world. And the fundamental aspects behind its therapeutic benefits are very poorly understood, and progress in this regard has been further hindered by a consistent discrepancy between traditional theory and scientific explanations. There is no an ideal evaluation criteria and standards for the optimal effect and intensity of stimulation of acupuncture till now, thus many acupuncturists only settle for inserting the needle into the acupoints but ignore the essentiality of achieving a certain intensity of stimulation and optimal effect of needle manipulation in the course of acupuncture treatment, leading to suboptimal or no therapeutic effect of acupuncture. Therefore, an understanding of the therapeutic mechanism of acupuncture and establishing methods and criteria for quantitative evaluation of* de qi* and the intensity and threshold of acupuncture could play an important role in the demonstration of validity and the wider use of this treatment modality in the world.

In the present study, 30 healthy adult volunteers were recruited to investigate intrinsic change in the body after* de qi* during acupuncture, such as change in local region of acupoints including acupoint blood flow, tissue displacement, electromyography, electroencephalography, and brain functional magnetic resonance imaging (fMRI).

## 2. Materials and Methods

### 2.1. Subjects and Procedures

The study was performed on 30 right-handed, 20–47 years old (29.0 ± 7.8), acupuncture naive healthy adult volunteers, 12 male and 18 female. The study was approved by the Ethics Committee of Tongji Medical College, HUST. For the quantitative evaluation of* de qi *during acupuncture, healthy adult volunteers (not patients) were recruited. The participants provide verbal but not written informed consent to participate in this study. The subjects were screened and those who had major medical illnesses, history of head trauma, neuropsychiatric disorders, use of medications within one week, and contraindications for exposure to high magnetic fields were excluded.

### 2.2. Procedures of Acupuncture

**Figure 1:**
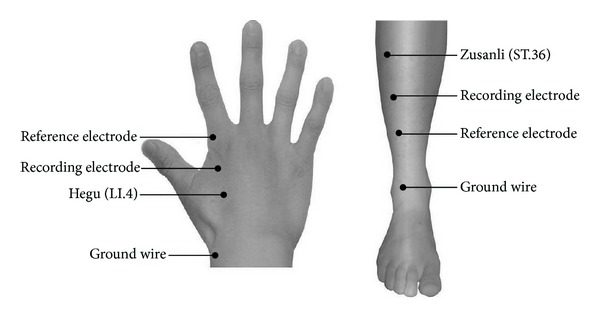
Acupoints schematic diagram. Acupuncture was performed using sterile disposable stainless steel needles at two acupuncture points on the right extremity in separate runs: ST.36 (traditionally known as the Zusanli acupoint) on the leg and LI.4 (traditionally, the Hegu acupoint) on the hand. The acupuncture point ST.36 is located in the tibialis anterior muscle, 4 fingerbreadths below the kneecap and 1 fingerbreadth lateral from the anterior crest of the tibia. The acupoint LI.4 is located in the dorsal surface of the web between the thumb and the index finger. Recording electrode and reference electrode are placed on the distal end of acupoints in the right limbs. Ground wires were placed on the dorsal surface to avoid electrical disturbance.

The subjects were instructed to lie still and keep their eyes closed during the procedure. Acupuncture was performed using sterile disposable stainless steel needles at different acupoints. The two acupuncture points on the right extremity in separate runs: ST.36 (traditionally known as the Zusanli acupoint) on the leg and LI.4 (traditionally, the Hegu acupoint) on the hand (seen in Figure 1). The acupoint ST.36 is located in the tibialis anterior muscle, 4 fingerbreadths below the kneecap and 1 fingerbreadth lateral from the anterior crest of the tibia. The acupoint LI.4 is located in the dorsal surface of the web between the thumb and the index finger. These two acupoints were chosen because of their easy accessibility of* de qi* sensation and were most frequently used in acupuncture. Although several reports indicated that there was a difference in acupoints and nonacupoints when acupuncture was administrated, in the present study we only focus on the quantitative evaluation of* de qi* and the relationship between* de qi* degree and the clinical therapeutic effects.

**Figure 2:**
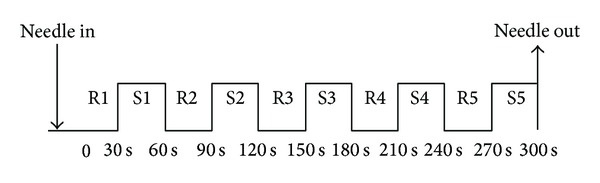
Acupuncture manipulation paradigm. Manual acupuncture was administered to LI4 and ST36 on the right. The subject's sensitivity to needling was pretested and adjusted to tolerance prior to scanning. After remaining in place for 30 s (R1), the needle was rotated forward and backward with stimulation for 30 s at the rate of 60 times per minute with an amplitude of approximately 180° in each direction (S1). After a rest period of 30 s (R2), needle manipulation was repeated in the same manner (S2). The needle was withdrawn after completion of 5 cycles of R-S acupuncture.

Disposable sterile stainless steel needles (KINGLI Medical Appliance Co., Ltd., Wuxi, China) of 0.22 mm in diameter and 40 mm in length were used. The needle was inserted vertically to a depth of 2-3 cm. The sensitivity of the subject to needle manipulation was tested and adjusted to tolerance prior to procedure, aiming to elicit* de qi* sensation without noxious pain. In the event of a sharp painful sensation, the needle position should be readjusted and the pain would disappear within a few seconds. During the acupuncture procedure, the subjects were questioned about the sensations that they had felt and whether the* de qi* sensations (aching, pressure, soreness, heaviness, fullness, warmth, cooling, numbness, tingling, and dull pain), sharp pain or any other sensations, occurred. The stimulation paradigm is depicted in Figure 2. The needle was kept in place for 2 min prior to needle manipulation and then was rotated approximately 180° in each direction with even motion at the rate of one cycle per second, which is a technique used in clinical practice. The two stimulation blocks, for example, S1 and S2, were separated by an interval of 30 s as a break period with needle remaining in place.

### 2.3. Tissue Displacement

In vivo ultrasonic imaging using a System FiVe (GE-Vingmed) at 7.5 MHz was performed on the healthy subjects at different stages of acupuncture needle stimulation including before* de qi* and during* de qi*. Displacements were estimated using the ultrasonic radio-frequency (RF) data, with a 2 mm window and a window overlap of 60%. Seventy RF scans were acquired continuously during each experiment at the rate of 13.2 frames per second. Ciné-loop displacement images were generated off line during and between the different stages of acupuncture stimulation.

### 2.4. Laser Doppler Perfusion Imaging (LDPI)

A PeriScan PIM II LDPI (made by Perimed Company, Sweden) was used in this study with a scanning laser wavelength of 670 nm and a maximum output power of 1 mW. An NR scanning pattern was used, of minimum scanning accuracy, with usual sampling points at 35 (width) × 40 (height) and an image with a pixel size of 0.5 × 0.5 mm^2^ [2]. LDPI 2.5 imaging software was used for recording, storage, analysis, and processing of the acupoint blood perfusion image.

### 2.5. Electromyography (EMG)

The electromyography system (Viking Quest, NICOLET, USA) was used to perform nerve stimulation and reflex recording, before and after* de qi* induced by acupuncture treatment at Hegu and Zusanli acupoints. The skin resistance overlying acupoints was made as minimal as possible by shaving the area and brushing it with alcohol. After skin preparation with 70% alcohol, disposable silver-silver chloride pregelled snap on electrodes (9 mm diameter recording surface) were placed 15 mm proximal and distal to acupuncture point, in parallel with the muscle fibers, for maximum selectivity and sensitivity. The stimulator was placed over the distal to the peroneal nerve and median nerve. A reference ground electrode was placed over the medial epicondyle or phalange of index finger, respectively, as recommended by EMG protocol. The EMG machine worked in conjunction with a Pico Technology Limited ADC-100 dual channel oscilloscope, which connected the EMG machine to a laptop computer. Pico Log data logging software was used to collect and analyze the EMG data. The program was set at a sampling rate of 1000 Hz over the 10 seconds testing time. The volunteers were instructed to keep the arm resting on the couch throughout.

### 2.6. Electroencephalography (EEG)

EEGs constitute an objective, continuous, noninvasive, and simple method for evaluating cerebral functions. In the present study, the EEG system, manufactured by Biopac Systems, Inc., was used on EEGs during acupuncture stimulation. EEGs were collected from 12 channels, and frequency bands with *α*-waves (8–13 Hz), *β*-waves (13–30 Hz), *θ*-waves (4–8 Hz), and *δ*-waves (0.5–4 Hz) as standards were used. EEGs consist mainly of *α*-waves and *β*-waves. It is abnormal if EEG exhibits slow waves in the waking stage. The region of the brain per electrode was marked as frontal, parietal, temporal, and occipital. The experimental subjects' EEGs were measured through the electrodes via the 10–20 electrode placement method. Electrodes were attached to both ears as reference electrodes.

### 2.7. Functional Magnetic Resonance Imaging (fMRI)

Brain imaging was conducted on a 1.5-T Siemens Sonata MRI system equipped for echo planar imaging (EPI) with a standard head coil. Functional scans were collected with sagittal sections parallel to the AC-PC plane, slice thickness 3.0 mm with 20% gap. Imaging encompassed the entire brain, including the cerebellum and brainstem. The functional data were acquired by a T2*-weighted gradient echo sequence (TE 30 ms, TR 4 s, matrix 64 × 64, FOV 200 mm, flip angle 90°, in-plane resolution 3.125 × 3.125 mm). A set of 3D MPRAGE (magnetization-prepared rapid acquisition gradient echo) images, voxel size of 1 mm^3^, 128 images per set, and a set of T1-weighted high-resolution structural images (TE 3.39 ms, TR 2.73 s, matrix 192 × 256, FOV 256 mm, flip angle 7°, in-plane resolution 1 × 1 mm, slice thickness 1.33 mm) were acquired prior to functional scans.

### 2.8. Statistical Analysis

SPSS 13.0 software for Windows (SPSS Inc., USA) was used for statistical analysis. Continuous variables were expressed as mean ± S.D. The group comparison was performed with two-tailed* t*-test and SNK method (ANOVA). The *P* values of less than 0.05 were considered to be statistically significant.

## 3. Results

Acupuncture increased tissue displacement and skin blood flow on acupoints after* de qi*.

**Figure 3:**
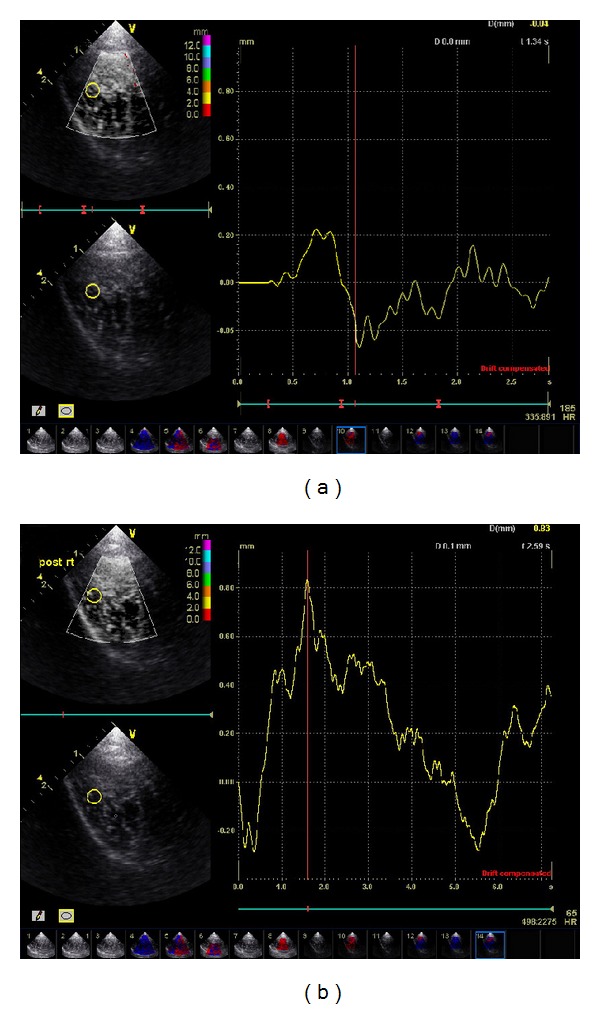
Tissue displacement on acupoints following needle stimulation before and after* de qi*. In vivo ultrasonic imaging using a System FiVe (Vingmed) at 7.5 MHz was performed on the healthy subjects at different stages of acupuncture needle stimulation including before* de qi* and during* de qi*. Displacements were estimated using the ultrasonic radio-frequency (RF) data. Seventy RF scans were acquired continuously during each experiment at the rate of 13.2 frames per second.

It was found that soft-tissue displacement could be estimated using only the stimulus caused by the movement of the needle. In the present study, the amount of tissue displacements, induced by acupuncture treatment, was measured by in vivo ultrasonic imaging before* de qi* and during* de qi* stage. As shown in Figure 3, the distance of tissue displacements in Zusanli was found to increase in amplitude by up to 0.167 ± 0.047 mm during* de qi* stage (Figure 3(b)), compared with 0.105 ± 0.027 mm, the distance before* de qi* (Figure 3(a), *P* < 0.01).

**Figure 4:**
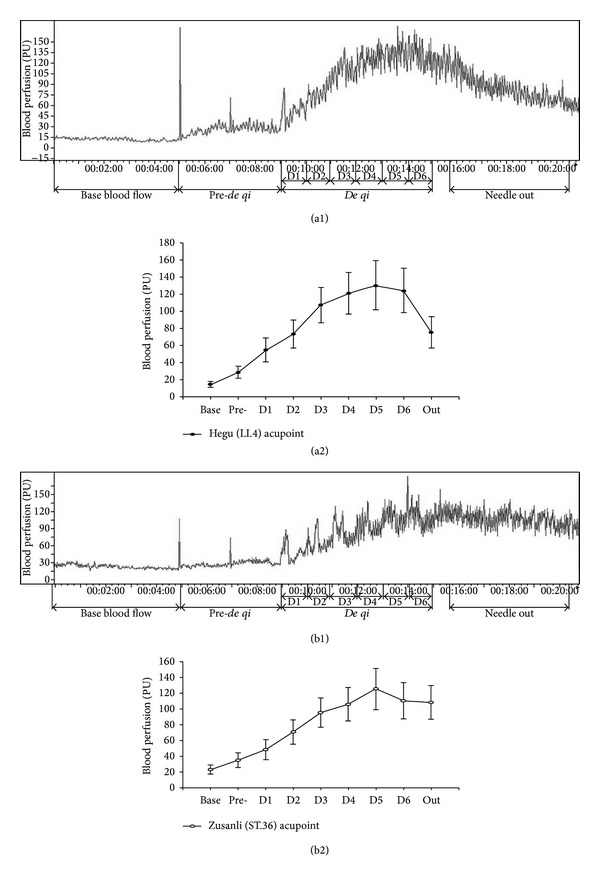
Blood flow changes at acupoints following acupuncture before and after* de qi*. A PeriScan PIM II laser Doppler perfusion imaging (LDPI) was used in this study for analysis and processing of the acupoint blood perfusion image. Before the acupuncture needle stimulation, the basal blood flow of the healthy subjects was low and then significantly increased when deep-punctured but without* De qi* (*P* < 0.05). When the healthy subject felt* de qi* sensation, the skin blood flows at the acupoints were more evident than those before* de qi* (*P* < 0.01) and these changes were time-dependent (Figures 4(a1) and 4(a2) for Hegu and Figures 4(b1) and 4(b2) for Zusanli).

In addition, change of blood flow in Hegu and Zusanli acupoints was determined by the LDPI technology. We found that blood flow increased transiently when the acupuncture needle was inserted into the acupoint Hegu and then reverted to baseline before* de qi*. When the volunteers felt the sensations of numbness, heaviness, distention, and soreness, representing* de qi*, the skin blood flow increased significantly and was maintained at a relatively high level for up to 6 min (D1–D6, Figures 4(a1) and 4(a2)). Similar results were also found in Zusanli acupoint (see Figures 4(b1) and 4(b2)).

Acupuncture increased amplitude of myoelectricity after* de qi*, although no remarkable change was seen in EEG.

**Figure 5:**
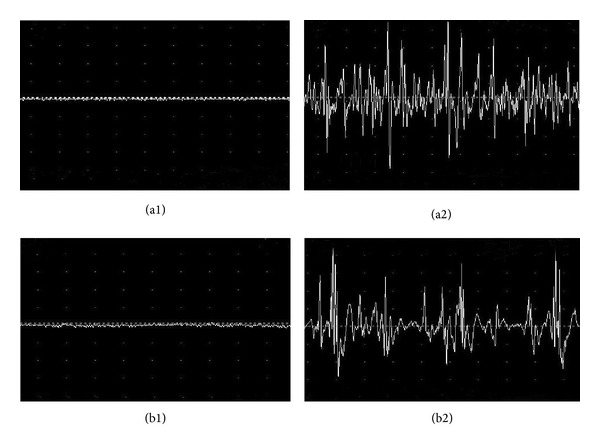
Myoelectricity and deep resistance at acupoints following acupuncture before and after* de qi*. The Viking Quest portable EMG/evoked potential systems were used in this study for analysis of the myoelectricity and deep resistance. The deep resistance before* de qi* in Hegu acupoint of the healthy subjects was 34.85 ± 12.43, which was increased to 51.98 ± 11.84 uV after* de qi* (*P* < 0.01). The similar results were seen for Zusanli acupoint which was 39.38 ± 9.07 uV before* de qi* and 55.18 ± 6.19 uV after* de qi* (*P* < 0.01).  Figure 5 is the representative image of myoelectricity at acupoints following acupuncture before and after* de qi* ((a1) and (a2) for Hegu; (b1) and (b2) for Zusanli). The amplitudes of myoelectricity after* de qi* in Hegu and Zusanli were significantly increased than those before* de qi*.

The Viking Quest portable EMG/evoked potential systems were used in this study for analysis of the myoelectricity and deep resistance. The deep resistance before* de qi* in Hegu acupoint of the healthy subjects was 34.85 ± 12.43 uV, which was increased to 51.98 ± 11.84 uV after* de qi* (*P* < 0.01). The similar results were seen in Zusanli acupoint, which were 39.38 ± 9.07 uV before* de qi* and 55.18 ± 6.19 uV after* de qi* (*P* < 0.01). Figures 5(a1), 5(a2), 5(b1), and 5(b2) are the representative image of myoelectricity in Hegu acupoint following acupuncture before and after* de qi*. The amplitude of myoelectricity after* de qi* in Hegu was significantly increased than that measured before* de qi*.

**Figure 6:**
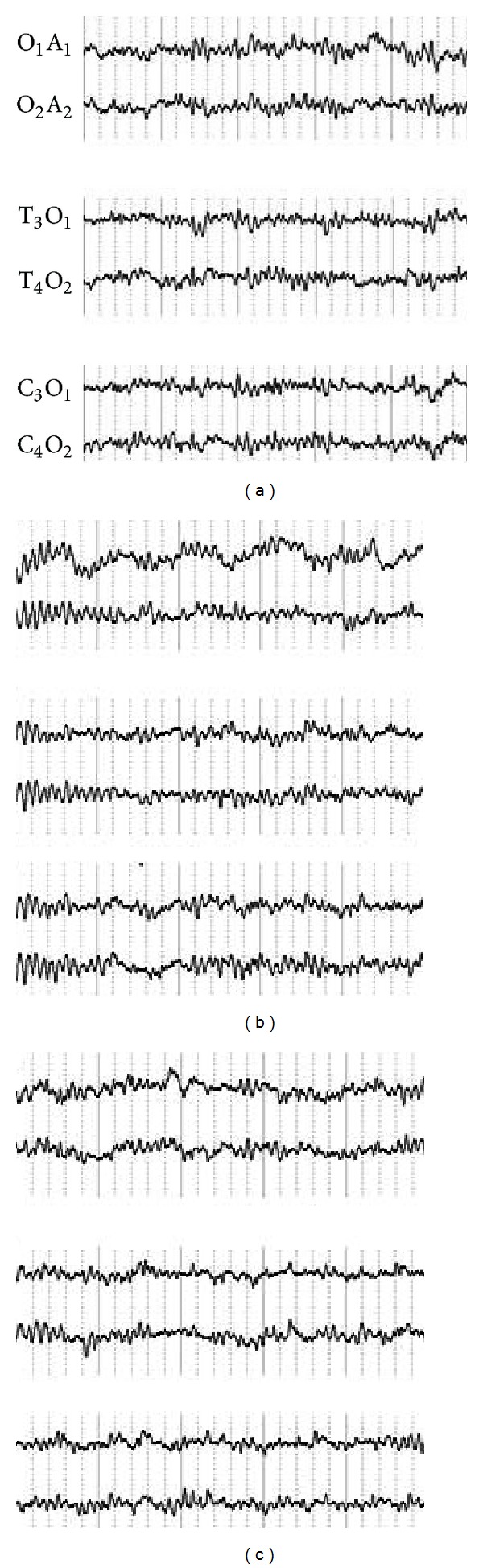
Change of electroencephalogram in Hegu acupoint following acupuncture before and after* de qi*. O-A, T-O, and C-O represent the electrodes placed for the different brain regions. We found that in different brain regions, the change of electroencephalogram before and after* de qi* was not evident.

In addition, the change of electroencephalogram in Hegu acupoint following acupuncture before and after* de qi* was determined. In Figure 6, O-A, T-O, and C-O represented the electrodes placed in the different brain regions. We found that in different brain regions, the change of electroencephalogram before and after* de qi* was not evident.

Acupuncture treatment induced fMRI signal increase/decrease in different brain regions on Zusanli acupoint before and after* de qi*.

**Figure 7:**
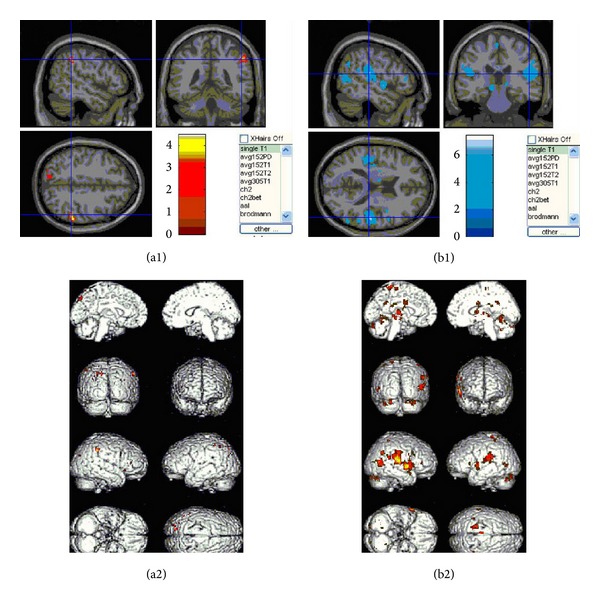
Change of functional magnetic resonance imaging (fMRI) in Zusanli acupoint following acupuncture before and after* de qi*. Mean results of functional MR images of brain activation/deactivation of nine subjects in each stimulation paradigm. Representative color-coded statistical maps derived from data obtained during the four stimulations paradigms (overlaid on morphologic MR images) show the distribution of foci with significant increases (shown in the spectrum from red to yellow) and decreases (shown in the spectrum from blue to green) in signal intensity, relative to that of the respective states.

The results for fMRI (Figure 7; Tables 1 and 2) during acupuncture at ST.36 showed an activation/deactivation pattern in the different brain regions. Representative color-coded statistical maps derived from data obtained during the four stimulations paradigms (overlaid on morphologic MR images) showed the distribution of foci with significant increases (shown in the spectrum from red to yellow) and decreases (shown in the spectrum from blue to green) in signal intensity, relative to that of the respective states.

Multiple regions of signal increase were observed during acupuncture needle manipulation. Acupuncture-induced activation over the ipsilateral inferior parietal lobule (Brodmann areas 40), ipsilateral subcortex white matter, ipsilateral superior temporal gyrus (Brodmann areas 22), ipsilateral gyrus frontalis medius (Brodmann areas 47), ipsilateral prefrontal lobe (Brodmann areas 46), ipsilateral cuneate lobe (Brodmann areas 19), ipsilateral posterior central gyrus (Brodmann areas 3), the contralateral precuneus (Brodmann areas 7), the contralateral inferior parietal lobule (Brodmann areas 40), the contralateral central occipital gyrus (Brodmann areas 19), the contralateral frontal lobe frame gyrus (Brodmann areas 10), and the contralateral supramarginal gyrus (Brodmann areas 40). In addition, there was activation in the ipsilateral ventriculus dexter cerebri and mesencephalon.

Acupuncture needle manipulation related deactivation (signal intensity decreased during* de qi* stage as compared with that before* de qi*) was found bilaterally in the majority of structures including posterior central gyrus (Brodmann areas 2 and 3), putamen, inferior parietal lobule (Brodmann areas 40), culmen cerebelli, intercerebral fissure, clivas, thalamus, cingulate gyrus (Brodmann areas 24), and occipital lobe (Brodmann areas 18 and 19). In addition, deactivation also occurred in the contralateral insular lobe, the contralateral mesencephalon, the contralateral subthalamic nucleus, the ipsilateral superior temporal gyrus (Brodmann areas 22 and 52), the ipsilateral gyrus frontalis medius (Brodmann areas 6, 9, and 45), the ipsilateral dentate body of cerebellum, the ipsilateral corpus callosum, and the ipsilateral midtemporal gyrus (Brodmann areas 21).

## 4. Discussion

Acupuncture has been widely used for a range of acute and chronic disorders in China, and during recent decades acupuncture has been used in many countries around the world. In spite of popular clinical applications, there are no objective evaluation criteria for optimal effect of needle manipulation, the intensity of stimulation during acupuncture. And evidence to support the use of acupuncture needs to be established. In fact, it has been accepted that induction and occurrence of* de qi* are a prerequisite for acupuncture and often an indicator of a clinical acupuncture effect [4, 6, 7, 9].* De qi*, which manifests as numbness, heaviness, distention, and soreness, with spreading sensation and manifests as heavy and tight sensation coming from beneath the needle, is the sine qua non of acupuncture for the achievement of a clinical therapeutic effect according to traditional Chinese medicine. And most of the experts in acupuncture consider that* de qi* is the intensity threshold of acupuncture treatment by means of needle rotation, upright and down, and acupuncture exerts its clinical therapeutic effects only under the certain condition of achieving* qi *(*de qi*). In spite of the importance and necessity of* de qi*, there is a lack of adequate experimental data to indicate what the intrinsic property of* de qi* is and how to evaluate it quantitatively. Till now, there are no ideal objective evaluation methods and criteria for optimal effect of the needle manipulation and for the intensity of stimulation. Many acupuncturists only acquire acupuncture needle to stick into the acupoints but ignore the essentiality of acupuncture intensity and threshold in the course of acupuncture treatment, leading to the impairment of the therapeutic effect of acupuncture.

In our study, it was a surprise to find that* de qi* induced intrinsic change in the human body during acupuncture treatment, including changes in acupoint blood perfusion, tissue displacement, electromyography, electroencephalography of local region at acupoints, and brain fMRI signals. The results showed that needle stimulation after* de qi* significantly increased blood perfusion, tissue displacement, and the amplitude of myoelectricity in the acupoints. Furthermore, acupuncture treatment induced brain fMRI signal increase/decrease in different brain regions although no significant change was seen in electroencephalogram. The intrinsic changes indicated that* de qi* elicited intense response of human body to acupuncture, especially at the location of acupoints and even in the brain, and it could be evaluated quantitatively, which might shed light on the therapeutic mechanism of acupuncture and facilitate the wider use of this treatment modality in the world.

In our report, the amount of tissue displacements, induced by acupuncture treatment, was measured by in vivo ultrasonic imaging before* de qi* and during* de qi*. Tissue displacements were found to significantly increase in amplitude after* de qi* compared to those before* de qi*. Tissue displacement caused by acupuncture needle manipulation following needle rotation may deliver a mechanical signal into the subcutaneous tissue and consequently generate important effects on cellular elements (fibroblasts, blood vessels, and sensory nerves) present within this tissue [1]. This may prove to be the key to acupuncture's therapeutic mechanism and the proposed imaging technique, the key method for monitoring this effect.

In addition, changes of blood flow at acupoints Hegu and Zusanli were determined by the LDPI technology. In our study, the local response could be seen clearly around the acupoint; the increment of blood perfusion was higher (about 10%) around Hegu and Zusanli after* de qi* than that measured before* de qi*, and the increase of blood perfusion was maintained at a relatively high level. The local increase of blood flow may be caused by axon reflection. An acupuncture-induced neural signal can be reflected along the branch of the same axon to the skin surface and can cause the release of substance *P*, which further evokes the release of histamine from nearby mast cells and causes vasodilatation and increase of blood perfusion [10]. The increase of blood perfusion and vasodilatation might be explained by the finding that several volunteers felt the sensation of heat and perspiration during acupuncture manipulation, which indicated* de qi*.

Furthermore, electrophysiological changes induced by* de qi* of acupuncture were also determined in this study. We found that acupuncture after* de qi* significantly decreased the deep electrical resistance at the acupoints and increased the amplitude of myoelectricity, suggesting that the local tissue of acupoints responded to the needle acupuncture. However, the change of electroencephalogram in different brain regions before and after* de qi* was not evident. The EEG results were consistent with those of Starr et al. [11] but appear to be contradictory with other reports in the literature [12–15]. Indeed, this is not entirely surprising in view of the difficulties in the measurements being attempted and the techniques adopted. During the recording of a normal EEG the signal may vary considerably and the effect of acupuncture has to be found within these variations [16]. In addition, the magnitude of the changes brought about by acupuncture may be small and conventional paper recording of the EEG is unlikely to be sufficiently sensitive to demonstrate changes [16]. Digital EEG recordings and brain mapping techniques which allow more quantitative data analysis offer better prospects in this regard.

Finally, we investigated the fMRI signal change induced by the acupuncture treatment and found that different brain regions were activated or deactivated in response to the needle stimuli. Acupuncture with* de qi* resulted in a marked predominance of signal attenuation or deactivation in the posterior central gyrus, putamen, inferior parietal lobule, thalamus, cingulate gyrus, occipital lobe, insular lobe, subthalamic nucleus, superior temporal gyrus, gyrus frontalis medius, midtemporal gyrus, and cerebellum. On the other hand, clusters of activated regions were seen in the inferior parietal lobule, subcortex white matter, superior temporal gyrus, ipsilateral gyrus frontalis medius, prefrontal lobe, ipsilateral cuneate lobe, ipsilateral posterior central gyrus, contralateral precuneus, inferior parietal lobule, central occipital gyrus, frontal lobe frame gyrus, and supramarginal gyrus. During acupuncture manipulation, several cortical and subcortical areas of human brains responded, according to the previous studies of acupuncture at ST36, which were localized at thalamus, insula, cingulategyrus, temporal gyrus, and cerebellum [17–21]. However, there are some different activated areas, such as basal ganglia and PAVN. Our results further validated findings of those previous studies. Acupuncture at analgesic acupoints, such as LI4 (*Hegu*) and ST36 (*Zusanli*), can modulate the hypothalamus and limbic system, which are pain-related neuromatrices [2, 17, 20, 22–24]. According to TCM, all acupoints are located along the meridians. ST36 is a commonly used acupoint on the stomach meridian of foot-*Yangming,* which starts from the lateral side of the ala nasi, ascends to the ipsilateral forehead, and descends to the dorsum of the foot, with a branch extending to the tip of the great toe [25]. The activation of the ipsilateral middle frontal gyrus is in accordance with the TCM theory. This area of the brain includes the pathway of* qi *along the stomach meridian of foot-*Yangming*. In the aspect of curative effects, the stomach meridian of foot-*Yangming *has a therapeutic effect for mental problems, gastralgia, and intestinal pain [25]. Our preliminary study demonstrated that* de qi* elicited significant response to acupuncture in the specific brain regions, but the mechanisms whether different acupoints are coupled with specific brain regions are not clear, and the reasons that the specific brain regions responded to* de qi* intensively also remain to be elucidated.

## 5. Interpretation

In summary, we have shown that acupuncture with* de qi* elicited the intrinsic change of human body. These recordings of all the aspects above may be taken as an indicator of* de qi* sensation. Biochemical effects shown by tissue displacement and blood flow change after acupuncture manipulation were derived from mechanical stimulation of connective tissue and potential spreading of these effects along connective tissue planes, which might deliver a mechanical signal into the subcutaneous tissue, induce the release of several pain-related substances, and then extend the communicated signal spreading. These processes might consequently enhance the response of specific brain regions and trigger the nerve-immune-secretion network to alleviate pain. Ongoing investigations over a larger pool of human subjects correlating with biochemical and neurological as well as morphological effects are expected to shed important light on the therapeutic mechanism of acupuncture and facilitate the intensive propagation of this treatment modality in the world.

## Figures and Tables

**Table 1:** Activated regions in fMRI after *de qi* in Zusanli acupoint following acupuncture.

Anatomy	BA	Side	Talairach			*Z* score
			*X* (mm)	*Y* (mm)	*Z* (mm)	
Inferior parietal lobule	40	R	60	−38	42	3.93
Subcortex white matter		R	22	−66	28	3.71
			32	−52	38	3.02
Precuneus	7	L	−24	−80	44	3.22
			−12	−76	46	3.06
Superior temporal gyrus	22	R	30	12	−22	2.91
Inferior parietal lobule	40	L	−46	−58	52	2.86
			−52	46	54	2.57
Gyrus frontalis medius	47	R	36	22	−6	2.37
			48	36	10	2.74
Central occipital gyrus	19	L	−30	−76	48	2.74
Prefrontal lobe	46	R	44	38	18	2.68
Cuneate lobe	19	R	16	−80	20	2.62
Frontal lobe frame gyrus	10	L	−40	40	24	2.59
Posterior central gyrus	3	R	18	68	60	2.55
Ventriculus dexter cerebri		R	2	8	14	2.46
Mesencephalon		R	4	−38	−20	2.44
Supramarginal gyrus	40	L	−38	−52	34	2.42

Table 1 lists the Talairach coordinates. Numbers in cortical areas of the images correspond to Brodmann areas. Multiple regions of signal increase were observed during acupuncture needle manipulation of the right leg at ST.36. Acupuncture induced activation over the ipsilateral inferior parietal lobule (Brodmann areas 40), ipsilateral subcortex white matter, ipsilateral superior temporal gyrus (Brodmann areas 22), ipsilateral gyrus frontalis medius (Brodmann areas 47), ipsilateral prefrontal lobe (Brodmann areas 46), ipsilateral cuneate lobe (Brodmann areas 19), ipsilateral posterior central gyrus (Brodmann areas 3), the contralateral precuneus (Brodmann areas 7), the contralateral inferior parietal lobule (Brodmann areas 40), the contralateral central occipital gyrus (Brodmann areas 19), the contralateral frontal lobe frame gyrus (Brodmann areas 10), and the contralateral supramarginal gyrus (Brodmann areas 40). In addition, there was activation in the ipsilateral ventriculus dexter cerebri and mesencephalon.

**Table 2:** Deactivated regions in fMRI after *de qi* in Zusanli acupoint following acupuncture.

Anatomy	BA	Side	Talairach			*Z* score
			*X* (mm)	*Y* (mm)	*Z* (mm)	
Posterior central gyrus	2/3	L/R	−16	−42	68	5.63
			52	−24	18	5.33
Superior temporal gyrus	22/52	R	58	2	0	5.29
			58	10	−4	4.78
Putamen		L/R	−28	2	6	5.26
			−26	−8	8	5.04
			22	2	−2	3.99
Insular lobe		L	−45	22	18	4.55
			−36	−18	12	4.06
Inferior parietal lobule	40	L/R	−50	−34	22	5.14
			58	−42	22	4.03
			50	−44	24	4.00
Culmen cerebelli		L/R	8	−56	−8	4.72
			−2	−46	−10	4.27
Intercerebral fissure		L/R	−2	−62	0	4.21
			2	−34	22	3.36
Clivas		L/R	−2	−60	−18	3.51
			36	−70	−28	4.51
			26	−82	−28	4.06
			16	−86	−28	3.85
Gyrus frontalis medius	6	R	−2	−24	64	4.57
	9/45	R	48	6	30	3.99
			56	18	26	3.56
Dentate body of cerebellum		R	14	−58	−34	4.56
Mesencephalon		L	−10	−26	−8	4.45
Thalamus		L/R	−4	−12	2	3.63
			14	−28	0	3.32
Cingulate gyrus	24	L/R	4	0	28	4.08
			−12	0	34	3.86
Corpus callosum		R	2	−4	20	4.27
Midtemporal gyrus	21	R	54	−60	6	4.10
Occipital lobe	18/19	L/R	−52	−70	4	3.78
			34	−82	12	3.50
Subthalamic nucleus		L	−8	−14	−8	3.44

Table 2 shows the Talairach coordinates. Numbers in cortical areas of the images correspond to Brodmann areas. Deactivation was noted bilaterally in posterior central gyrus (Brodmann areas 2 and 3), putamen, inferior parietal lobule (Brodmann areas 40), culmen cerebelli, intercerebral fissure, clivas, thalamus, cingulate gyrus (Brodmann areas 24), and occipital lobe (Brodmann areas 18 and 19). In addition, deactivation also occurred in the contralateral insular lobe, the contralateral mesencephalon, the contralateral subthalamic nucleus, the ipsilateral superior temporal gyrus (Brodmann areas 22 and 52), the ipsilateral gyrus frontalis medius (Brodmann areas 6, 9, and 45), the ipsilateral dentate body of cerebellum, the ipsilateral corpus callosum, and the ipsilateral midtemporal gyrus (Brodmann areas 21).
